# Spondyloarthritis: How far are we from precision medicine?

**DOI:** 10.3389/fmed.2022.988532

**Published:** 2022-09-08

**Authors:** Jacqueline So, Ann-Sophie De Craemer, Dirk Elewaut, Lai-Shan Tam

**Affiliations:** ^1^Department of Medicine and Therapeutics, Prince of Wales Hospital, Hong Kong, Hong Kong SAR, China; ^2^Division of Rheumatology, Department of Internal Medicine and Pediatrics, Ghent University Hospital, Ghent, Belgium; ^3^Center for Inflammation Research, VIB-UGent, Zwijnaarde, Belgium; ^4^Department of Medicine and Therapeutics, The Chinese University of Hong Kong, Hong Kong, Hong Kong SAR, China

**Keywords:** spondyloarthritis precision medicine, genetics, microbiome, metabolomics, spondyloarthritis

## Abstract

Spondyloarthritis (SpA) is a family of heterogenous diseases consisting of different phenotypes. The exact disease mechanism remains unclear but evidence shows the complex pathophysiology with interplay between genome, microbiome, and immunome. Biologic DMARDs have markedly improved patients' disease control and quality of life. However, treatment response varies among patients. There is a growing need to identify biomarkers for the diagnosis, prognosis, prevention, and treatment of SpA. Genomic studies have been the research focus in the past two decades and have identified important genes involved in SpA. In recent years, emerging evidence supports the link between gut and joint inflammation in SpA, in which the role of gut microbiome in SpA is of great interest. Herein, potential genetic and gut microbial biomarkers for predicting treatment response are discussed. Novel strategies targeting dysbiosis in SpA are also summarized. These results represent a significant step toward precision medicine for patients with SpA.

## Introduction

Spondyloarthritis (SpA) is a group of seronegative inflammatory diseases characterized by spondylitis, peripheral arthritis and enthesitis. Axial spondyloarthritis (axSpA) is the prototypical form of SpA which includes non-radiographic axSpA and radiographic axSpA [also known as ankylosing spondylitis (AS)]. Other diseases in this family include psoriatic arthritis (PsA), reactive arthritis, enteropathic arthritis related to inflammatory bowel diseases (IBD) and undifferentiated SpA. Diseases in the SpA family share common clinical features including sacroiliitis, peripheral arthritis, enthesitis and dactylitis. Some patients may also have extra-musculoskeletal manifestations such as uveitis, IBD and psoriasis. Currently, the management of SpA is based on disease subtypes and manifestations. Non-steroidal anti-inflammatory drugs (NSAIDs) have been the mainstay of treatment for spondylitis for decades. However, the efficacy of NSAIDs in axSpA was suboptimal with 30–35% response rate only (1, 2). Majority of patients suffered from suboptimal disease control with persistent and debilitating back pain.

**Figure 1:**
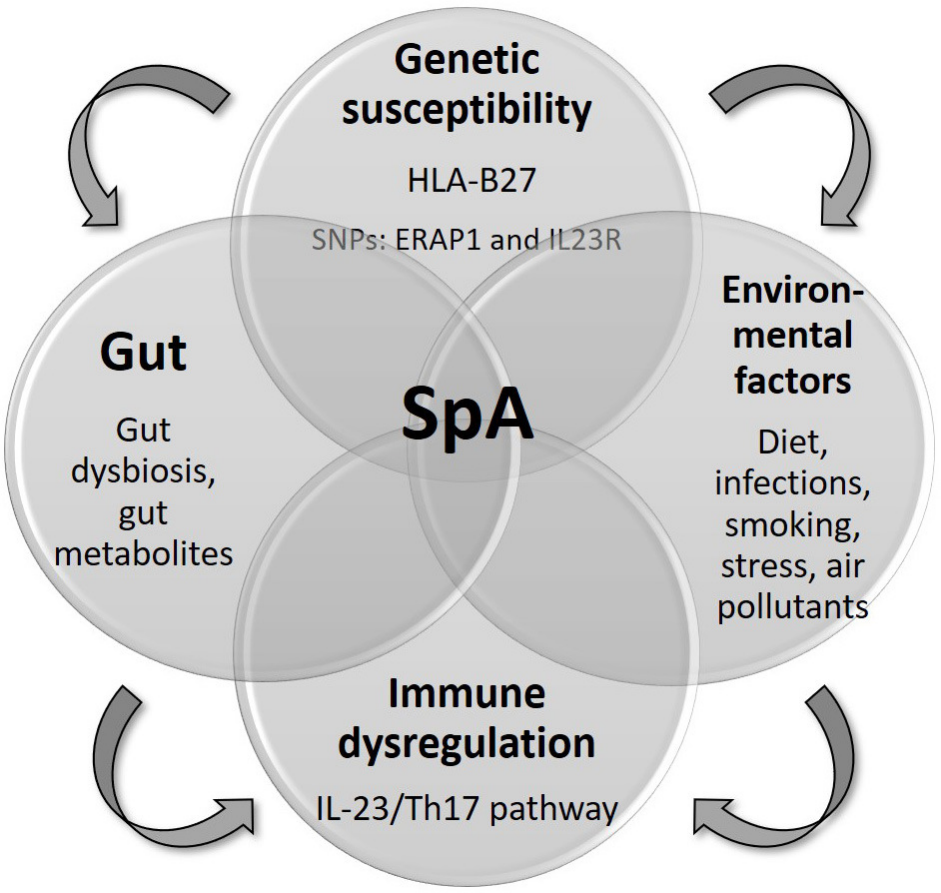
Pathogenesis of SpA.

The introduction of biologic disease modifying anti-rheumatic drugs (DMARDs), which target inflammatory cytokines or their receptors, was a major medical breakthrough in the treatment of SpA. Biologic DMARDs including anti-tumor necrosis factor (TNF), anti-interleukin (IL) 17 and anti-IL23 and targeted synthetic DMARDs have significantly improved treatment outcomes and the quality of life of SpA patients (3–5). However, there is not one drug that fits all patients. The current approach in choosing among biologics is mainly based on disease subtypes and concurrent manifestations such as monoclonal anti-TNF, but not recombinant TNF receptor- Fc fusion protein etanercept, for patients with concomitant axSpA and uveitis and anti-IL17 or anti-IL23 for PsA patients with severe psoriasis (6, 7). Yet, clinical response to biologic DMARDs varies among patients. There is a substantial group of SpA patients who are poor responders or non-responders to biologic DMARDs (8). Also, clinical trials have showed that some biologics could be beneficial in one aspect of the disease while harmful or ineffective in another, for example, anti-IL17 might increase risk of exacerbation or even new onset of Crohn's disease and anti-IL23 is effective in treating PsA but not in axSpA (9, 10). These demonstrate the immunological heterogenicity in different disease subtypes. Many studies attempted to investigate the use of serum cytokines including IL-6, IL-17, IL-23, and TNF-α in predicting treatment response. However, the results were inconclusive (11). Twin studies showed that the concordance rates of AS in monozygotic and dizygotic twin were 50–75 and 20–27%, respectively (12, 13). This signifies that SpA is not entirely genetically determined, but is a result of the complex interplay of genetic and environmental factors (13) (Figure 1). With the medical advancement in SpA, are we ready to practice precision medicine according to molecular phenotyping such as genomics, metabolomics and microbiomics?

## Genetic factors and SpA

Human Leukocyte Antigen (HLA)-B27 is a Class I Major Histocompatibility Complex (MHC) molecule that strongly increases genetic susceptibility to SpA and accounts for one-fifth of the AS heritability (14), and to a lesser extent PsA. The pathogenesis of SpA remains uncertain but there are several hypotheses of HLA-B27 related SpA pathogenesis. First, antigen peptide processing generates highly immunogenic peptide- MHC complexes that trigger T-cell immune response. Second, HLA-B27 free heavy chain homodimers may become targets for natural killer cells. Third, misfolding of HLA-B27 in the endoplasmic reticulum (ER) results in activation of an unfolded protein response and the production of IL-23(15, 16). The prevalence of HLA-B27 in axSpA patients varies among different ethnicities. Around 70–80% of Caucasian patients with axSpA are positive for HLA-B27 while HLA-B27 is only present in 20–60% of axSpA patients in Latin America, Middle East, and Japan (17–19). Animal model in the early 90s showed that HLA-B27/β_2_ microglobulin transgenic rats with high transgene copy numbers developed spontaneous spondyloarthritis-like phenotype while those with low transgene copy numbers remain healthy (20). This showed that the effect of one gene itself is not sufficient to cause SpA.

Genome-wide association studies (GWAS) have identified 113 single nucleotide polymorphism (SNP)s that contribute to disease susceptibility for AS including two major discoveries: Endoplasmic reticulum aminopeptidase 1 (ERAP1) variant and IL23-receptor (IL23R) (21, 22). ERAP1 encodes the aminopeptidase involved in trimming peptides to the optimal length in order to be presented on MHC Class I molecules in the ER, which is a key molecular mechanism involved in AS (23). *In vivo*, inhibition of ERAP1 also reduced the expansion of Th17 and production of IL-2 and IL-17A (24). However, further clinical studies are needed to prove the therapeutic role of ERAP1 inhibition in axSpA. Identification of IL-23R, a gene involved in the IL-23/Th17 axis, is another important finding in GWAS (23). IL-23 is a pro-inflammatory cytokine that drives the differentiation of Th17 lymphocytes, which in turns produce IL-17 and other proinflammatory cytokines involved in SpA. Animal studies showed that overexpression of IL-23 could induce SpA features (25). The important role of the IL-23/Th17 axis in SpA has been vastly supported by genetic studies, animal models, and therapeutic trials (26). Other overlapping genes involved in the IL-23/Th17 pathway for axSpA and PsA include IL-12B, STAT3, and CARD9.

Could genotyping guide therapeutic choices in SpA? A systematic review on genetic variants in relation to treatment response showed inconclusive results (27). Polymorphisms of the TNF receptor superfamily 1A and 1B (TNFRSF1A/1B) gene were most commonly investigated to predict treatment response of anti-TNF in SpA (Table 1). The study conducted by Seitz et al. showed that TNF-α−308 G/G genotype was associated with better response to anti-TNF compared to patients with A/A or A/G genotype in AS and PsA patients (28). Another study in Italy also found that TNFα−308 A allele and IL-6−174GG homozygosis were good predictors of drug-survival of the first anti-TNF in SpA (29). However, Murdaca et al. did not find a positive correlation between TNF-α−308 or−238 genotype and anti-TNF treatment response in PsA (33). Another two studies in AS did not confirm the association between TNF-α−308 or-238 and anti-TNF treatment response neither (30, 31). Whereas, a study of 106 AS patients in China found that TNF-α−857 C/C genotype and TNF-α−1031 T/T genotype were associated with better treatment response to anti-TNF therapy (30). *TNFAIP3* polymorphism rs6920220 and rs610604 were associated with better quality of life after 3 months of anti-TNF use in PsA (32). SNP + 489 GG and GA genotype of the TNF-α gene may predict good treatment response to etanercept in PsA (33). Apart from TNF receptor family, V allele of the SNP rs396991 (V158F) in FCGR3A gene showed better response to infliximab (anti-TNF) at 6 months in axSpA (34). A recently published pilot study showed that 12 interferon regulated genes might predict magnetic resonance imaging (MRI) response to anti-TNF treatment in AS (35). There are studies that showed TNF polymorphisms may predict poor response to treatment too. Aita et al. showed that *TNFRSF1A* c.625 + 10A>G was associated with late response to infliximab in SpA (36) and TNFRSF1B polymorphism rs1061622 genotype was associated with non- response to a first-line anti-TNF treatment in AS (37).

**Table 1:** Studies on gene expression profiling for response prediction in SpA.

**References**	**Patient population**	**Drug treatment**	**Biomarker**	**Results**	**Definition of response**
Seitz et al. (28)	RA (*n* = 54), AS (*n* = 23), PsA (*n* = 10),	Infliximab, Adalimumab, Etanercept	TNF-α−308 G/G, A/G genotype	Patients with TNF-α−308 G/G genotype response better to anti-TNF in AS (*p* < 0.005) and PsA (*p* < 0.05)	AS: - Moderate response: an improvement of BASDAI ≥20 and ≤50% - Good response: BASDAI improvement of >50% - Non- responders: BASDAI improvement of <20%
					PsA: - Moderate response: DAS28 improvement ≥1.2 and ≤2.2 - Good response: DAS28 improvement >2.2.
Fabris et al. (29)	SpA (*n* = 187) Anti- TNF alpha never-switch (*n* = 123), anti-TNF alpha switchers (*n* = 64)	Infliximab, Etanercept, Adalimumab, Golimumab	TNFα−308 AA/AG vs. GG allele, IL-6−174GG homozygosis, NFR2, IL-6Rα, TGFβ and FCGR3A	TNFα−308 A allele (*p* = 0.007) and IL-6−174GG homozygosis (*p* = 0.035) were good predictor of 1st anti-TNF survival.	Good responder: BASDAI >50% decrease or change of ≥20
				NFR2, IL-6Rα, TGFβ and FCGR3A did not show association	
Tong et al. (30)	AS patients (106)	Infliximab or recombinant human TNF receptor-Fc	TNF*- α*-308 rs1800629, TNF- α-238 rs361525, TNF*- α*-1031 rs1799964 and TNF*- α*-857 rs1799724	TNF*- α*−857 C/C genotype (*p* = 0.0021) and TNF- α−1031 T/T (*p* = 0.0004) genotypes are predictors of good response	Poor responder: fulfilled ASAS20 Good responder: fulfilled ASAS40, 50 and 70 Non-responder: no improvement
Nossent et al. (31)	AS (*n* = 335)	Anti-TNF	TNF-α −238 A/G and −308 A/G	TNF-α −238 A (0 vs. 1%) and TNF-α −308 A allele were not associated with initial BASDAI response (*p* > 0.3)	–
Benito et al. (32)	PsA and PsO (*n* = 20)	Anti-TNF	TNFRSF1B, TNFRSF1A, TNFAIP3, TNIP1, TNF (-238), TRAF3IP2, TNF-308, TNF-857, TNF-1301	Two SNPs: rs610604 and rs6920220 (TNFAIP3) are associated with %EQ-VAS at 3 months	–
Murdaca et al. (33)	PsA (*n* = 57)	Etanercept, infliximab, adalimumab	SNPs−308 and−238 genotype	SNPs−308 and−238 genotype were not associated with clinical outcome of PsA after anti-TNF	Good responder: DAS28 improvement >1.2, Moderate responder DAS28 improvement >0.6– ≤1.2
				SNP + 489 GG and GA responded better than SNP + 489AA (*p* = 0.021).	
Morales-Lara et al. (34)	AS (*n* = 33), PsA (*n* = 16), RA (*n* = 41)	Infliximab	rs396991 polymorphism (V158F) in FCGR3A FF, FV, VV genotypes	V allele is associated with better response in AS at 6 months (*p* = 0.0034)	Responders: ACR improvement ≥20 and BASFI improvement ≥20
Harrison et al. (35)	AS (*n* = 26)	Anti-TNF	IFN – regulated genes (IRGs)	AS patients with a group of IRGs and lineage age) may be more susceptible to respond to TNF inhibitors by MRI outcome, but not ASDAS outcome	Responder: ASDAS ≥1.2 reduction
Aita et al. (36)	PsA (*n* = 82), AS (*n* = 55)	Infliximab (*n* = 32), Adalimumab (*n* = 38), Etanercept (*n* = 21), Golimumab (*n* = 4), Ustekinumab (*n* = 2), Secukinumab (*n* = 1)	TNFA polymorphisms (-1031T>C;-857C>T;-376G>A;-308G>A;-238G>A) and HLA-B27	TNFRSF1A c.625 + 10 A>G was associated with late response to anti-TNFα therapy (*p* = 0.031)	Responder: BASDAI ≤4 Non- responder: BASDAI >4 Early responder: respond <10 months
Schiotis et al. (37)	AS (*n* = 121)	Anti-TNF	SNPs	rs917997 in the IL18RAP gene (OR 3.35, 95% CI 1.388.15), rs755622 (OR 3.14, 95% CI 1.198.22) in the MIF gene, rs1800896 in the IL10 gene	Responder: BASDAI 50% improvement
				(OR 3.09, 95% CI 1.049.15), rs3740691 (OR 2.90, 95% CI 1.127.51) in the ARFGAP2 gene, rs1061622(OR: 2.46, 95% CI 1.006.04) in the TNFRSF1B gene were predictors of non-responder to 1st anti-TNF	

ASDAS, Ankylosing Spondylitis Disease Activity Score.

ACR 20/50/70, American College of Rheumatology 20/50/70% improvement.

BASDAI, Bath Ankylosing Spondylitis Disease Activity Index.

BASFI, Bath Ankylosing Spondylitis Functional Index.

DAS28, Disease Activity Score 28.

EQ-VAS, European Quality of life Visual Analog Scale.

Currently, there is no conclusive evidence showing that TNF polymorphism or other SNPs could predict clinical response to anti-TNF in SpA. However, different populations with various genetic compositions and environmental exposure were involved in different studies which may account for the non-replicable results. With the emergence of more biologic and targeted synthetic DMARDs, more studies are required to explore potential pharmacogenomic markers to guide personalized treatment.

## Gut microbiome and SpA

There is growing evidence supporting the pathogenic mechanism of the gut-joint axis in SpA (38). Mielants et al. performed ileocolonoscopic studies in patients with SpA in the 1980s and found that two-thirds of the patients with SpA had subclinical gut inflammation (39, 40). Clinical disease activity also correlated with the degree of gut inflammation (41). Those with chronic gut inflammation were also at higher risk of developing IBD (42). The GIANT (Ghent Inflammatory Arthritis and spoNdylitis cohort) confirmed these findings with nearly half of the patients with SpA showing subclinical gut inflammation; 6.5% of them developed clinically overt IBD over 5 years (43). The degree of bone marrow edema on sacroiliac joint MRI in patients with SpA also positively correlated with gut inflammation (44). These observations support the link between the gut and the joint in SpA.

Gut dysbiosis, an imbalance of microbiota, could increase gut permeability and trigger inflammatory responses, leading to auto-inflammatory diseases such as IBD and asthma (45, 46). Alterations in the gut microbiome are also observed in rheumatic diseases (47). HLA-B27 transgenic germ-free rats fail to develop arthritis and colitis (48). However, they develop disease phenotype after reintroduction of commensal bacteria (49). Also, SKG mice develop spondyloarthritis and colitis after intraperitoneal injection of curdlan (a major component of bacterial and fungal wall) (50). These animal findings underscore the importance of the gut microbiome and mycobiome in SpA. In humans, a recently published systemic review and meta-analysis showed a high inconsistency of the intestinal microbial composition in SpA reported in different studies (47). Costello et al. first identified a distinct microbial composition in the terminal ileum of AS patients, with an increased abundance of 5 families of bacteria (*Lachnospiraceae, Ruminococcaceae, Rikenellacea, Porphyromonadaceae*, and *Bacteroidaceae*) and a decreased abundance of 2 families of bacteria (*Veillonellaceae* and *Prevotellaceae*) (51). Later, Tito et al. found the abundance of genus *Dialister* in ileal biopsies correlated with Ankylosing Spondylitis Disease Activity Score (ASDAS) in SpA patients (52). Also, there was an increase in abundance of *Ruminococcus gnavus* (53) and a decreased abundance of anti-inflammatory *F. prausnitzii* A2-165 in fecal samples of SpA patients (54). Metagenomic sequencing of fecal samples of patients with AS in China showed an expansion of *Prevotella melaninogenica, Prevotella copri, Prevotella* sp. C561 and a relative reduction of *Bacteroides* spp, compared to healthy controls (55). Whereas, Manasson et al. showed an increased abundance of order Clostridiales and Erysioekotrichales and a decreased abundance of order Bacteroidales in fecal samples of PsA/SpA patients compared to healthy controls (56). The only study that specifically investigated gut microbiome in PsA showed a decreased abundance in *Akkermansia, Ruminococcus*, and *Pseudobutyrivobrio* in fecal samples of PsA patients, which resembles IBD dysbiosis (57).

Apart from bacterial microbiota, studies from IBD showed that fungal and viral microbiota are also important in regulating host immunity and maintaining gut homeostasis. A reduction of fungal diversity was observed in IBD patients (58). Also, the *Ascomycota* to *Basidiomycota* ratio was lowered in IBD patients compared to healthy individuals, with an expansion of *Candida albicans* and a reduction of *Saccharomyces cerevisiae*. Similarly, a pilot study showed distinct fecal mycobiota pattern in AS patients characterized by an increased abundance of *Ascomycota* (mainly the class of *Dothideomycetes*) and decreased abundance of *Basidiomycota* (mainly *Agaricales*) (59). Also, an increase abundanced of *Saccharomyces* in AS patients was associated with worse radiographic damage (59). In AS patients receiving biologic agents anti-TNF and anti-IL17 (56, 59), on top of bacterial dysbiosis, there were expansion of *Saccharomyetales* and *C. albicans* in post treatment fecal samples. Perturbation of gut virome was also observed in IBD patients. Studies showed an increased relative abundance of *Caudovirales bacteriophages* and *Microviridae bacteriophages* in fecal samples of IBD patients compared to healthy individuals (60). However, gut virome study in SpA is lacking. Future studies should comprehensively investigate the complex gut ecosystem including microbiome, mycobiome, and virome to improve our understanding of disease pathogenesis.

Perturbation in the gut microbial composition was observed in SpA. However, there is a great variability of microbiome profile between studies. This discrepancy could be related to different genetic backgrounds and environmental exposures. Also, sampling methods, DNA extraction protocol and analysis methods could affect the accuracy of the microbiome analysis. Confounding factors such as diet, antibiotic use and smoking should be taken into account in microbiome analysis. Further large- scale studies are needed to establish the SpA-specific, and ideally phenotype-specific, microbial communities. With the advance in sequence- based screening, metagenomic studies should be conducted which may provide the functional properties of SpA-specific microbes.

With the increasing association of gut dysbiosis and SpA, studies were also performed to identify potential microbial biomarkers to predict treatment response (Table 2). Manasson et al. investigated the change in gut microbiome in fecal samples of PsA/SpA patients after anti-TNF or anti-IL17 treatment, and found a distinctive microbial shift in patients receiving different treatments. The abundance of Clostridiales increased and Bacteroidales decreased after anti-TNF. In contrast, there was a significant reduction in abundance of Clostridiales and increased abundance of Bacteriodales after anti-IL17 (56). Perturbation in the fungal microbiota was also noted after anti-TNF and anti-IL-17 treatment, in particular an expansion of *Candida* and *C. albicans* after anti-IL17. Another pilot study of 20 AS patients also showed the dynamic variation of gut microbiome composition after anti-TNF treatment (61). There was an expansion in abundance of genus *Collinsella*, genus *Dialister*, genus *Escherichina-Shigella*, family *Actinomycetaceae*, family *Coriobactereriaceae*, family *Prevotellaceae* and a decrease in abundance of genus *Bacteriodes*, genus *Parasutterella* and family *Burkholderiaceae* and family *Lachnospiraceae* in AS fecal samples compared to healthy controls. The relative abundance of genus *Escherichina-Shigella* and *Klebsiella* positively correlated with Bath Ankylosing Spondylitis Disease Activity Index (BASDAI) while the relative abundance of *Lachnospiraceae* negatively correlated with BASDAI. This dynamic change in gut microbial abundance in association with disease activity suggests the potential use of some microbes as indicators for disease activity. Another study of 18 SpA patients showed that higher proportions of Burkholderiales in fecal samples at baseline could predict clinical response to anti-TNF at 3 months (62). Whereas, higher proportion of genus *Dialister* and genus *Salmonella* in fecal samples of SpA patients after 3 months of anti-TNF distinguished between good and poor responder to anti-TNF respectively (62). However, these studies were limited by small sample size and the lack of healthy controls in the latter one. Chen et al. showed that 6-month use of adalimumab (anti-TNF) restored gut microbiome in AS patients (63). Also, high abundance of *Comamonas* in fecal samples might predict poor response to adalimumab (63). Future studies are required to confirm the specific bacteria and their mechanism involved which contribute to treatment success or failure in SpA. Larger, longitudinal studies should be performed in order to validate the potential microbial markers in predicting treatment response.

**Table 2:** Studies on gut microbial abundances between responders and non-responders in SpA.

**References**	**Patient population**	**Treatment**	**Samples**	**Technology**	**Results**	**Definition of response**
Zhang et al. (61)	AS (*n* = 20)	Anti-TNF	Feces	16S rRNA sequencing	- Abundance of *Escherichina-Shigella* (*p* = 0.013) and *Klebsiella* (*p* = 0.022) positively correlated with BASDAI - Abundance of *Lachnospiraceae* negatively correlated with BASDAI	–
Bazin et al. (62)	SpA (*n* = 19)	Anti-TNF	Feces	16S rRNA sequencing	- Higher proportion of *Burkholderiales* in fecal samples at baseline could predict clinical response to anti-TNF at 3 months - Higher proportion of genus *Dialister* after 3 months of anti-TNF predict good responder to anti-TNF - Higher proportion of genus *Salmonella* after 3 months of anti-TNF predict poor responder to anti-TNF	No response: ASDAS ≤1 Partial response: ASDAS between 1 and 2 Clinical response: ≥2
Chen et al. (63)	AS (*n* = 30)	Adalimumab	Feces	16S rRNA gene sequencing	- Higher abundance of *Comamonas* in non- responder (*p*-value not available)	Clinical response: ASDAS >1 No response ASDAS ≤1

There is a growing interest in modulation of the gut microbiome composition in SpA by prebiotics, probiotics, antibiotics, dietary modifications or fecal microbiota transplantation (FMT) in order to restore the normal gut microbiota. Prebiotics long-chain inulin and fructooligosaccharides both effectively ameliorated colitis in HLA-B27/β_2_ microglobulin transgenic rats (64, 65). Administration of probiotic and prebiotic mixture containing *Lactobacillus acidophilus* La-5*, Bifidobactrium lactis* Bb-12 and inulin to HLA-B27/β_2_ microglobulin transgenic rats also significantly increased gut microbial diversity, stimulated growth of *Bifidobacterium animalis* and attenuated colonic inflammation (66). However, there is no clinical trial of prebiotic use in SpA patients and the use of probiotics to modify dysbiosis in AS patients is so far discouraging. A pilot study using the combination of probiotics *Lactobacillus Acdiophilus* and *Lactobacillus Salivarius* in eighteen quiescent ulcerative colitis patients with active SpA showed improvement in disease activity (67). However, another study using specific probiotics composed of two *Lactobacillus* and two *Bifidobacterium* did not show improvement of disease activity or bowel symptoms in patients with SpA (68). A randomized controlled trial (RCT) comparing the use of probiotic combinations of *Streptococcus salivarius, Bifidobacterium lactis* LAFTI B94, and *Lactobacillus acidophilus* LAFTI L10 with placebo in active SpA patients again failed to show its efficacy (69). The results of probiotic use in PsA were conflicting too (70, 71). The use of the antibiotic rifaximin has been studied in proteoglycan-induced AS mice and showed promising results in reducing AS disease severity, inhibiting production of inflammatory cytokines, and promoting growth of beneficial intestinal bacteria in the gut (72). Yet, future clinical studies on the use of antibiotics in treating SpA patients are required. FMT is effective in treating *Clostridium difficile* infection and IBD by restoration of normal gut microbiome composition. A case report showed resolution of peripheral arthritis in a PsA patient after FMT for Clostridium difficile infection (73). However, a RCT (the FLORA study) showed that the use of FMT was inferior to sham in treating peripheral PsA (74).

Diets could alter gut microbiota composition. Protective effects of dietary patterns and supplements in SpA have been studied (75). Low starch diet has been proposed in AS patients as high dietary starch might promote the growth of *Klebisella pneumoniae*, which is possibly associated with the development of AS, in the bowel (75). A longitudinal study of 35 AS patients showed improvement in disease severity with low-starch diet (76). Also, diary-free diet was found to be effective in lowering disease activity and reducing use of NSAID in a pilot study of AS patients (77). However, systemic review concluded that there were insufficient evidence showing the beneficial effects of low starch and dairy-free diet in AS patients (78). Mediterranean diet has anti-inflammatory properties. Recently published observational study by Ometto et al. showed greater improvement in disease activity in axial SpA patients receiving Mediterranean diet nutritional advice (79). Another cross-sectional observational study of 211 PsA patients showed low adherence to Mediterranean diet was associated with higher PsA activity (80). Nonetheless, more studies are needed to conclude the beneficial impact of specific diet pattern in SpA patients. On the other hand, 1,25 dihydrovitamin D 1,25 (OH)_2_D is involved in regulating the production of pro-inflammatory cytokines. Meta-analysis showed that lower vitamin D level was associated with higher disease activity in AS (81). However, the beneficial effect of vitamin D supplementation in AS is lacking. Whereas, limited data support the use of vitamin D in decreasing disease activity in PsA (82). Fish oil supplement marine n-3 polyunsaturated fatty acids (PUFA) have anti-inflammatory effects and could improve arthritis. A RCT showed high dose PUFA significantly reduced the use of analgesics in PsA patients although there was no significant improvement in disease activity (83). Yet, current evidence is insufficient to draw a clear conclusion on the effectiveness of gut microbiome modulation in SpA given the knowledge gaps in microbiome of SpA. With the increase in understanding of dysbiosis in SpA, tailored intervention on gut microbiota may hopefully reshape gut microbiota and bring SpA into a cure.

## Gut metabolomics and SpA

Various gut microbial metabolites including short chain fatty acids (SCFAs), tryptophan and vitamin B derivates are important in maintaining gut barrier integrity and immune response in AS (84). SCFAs such as acetate, propionate and butyrate, are generated by the bacterial fermentation of dietary fibers. SCFAs promote regulatory T cell (Treg) differentiation which in turn suppresses Th17 cell differentiation and reduces production of pro-inflammatory cytokines (84). The association of altered gut microbiota and reduction in beneficial fatty acids is observed in some immune- mediated diseases such as IBD and asthma (85, 86). Notably, butyrate is crucial in maintaining gut permeability through upregulation of tight junction protein (84). Dysbiosis with lower butyrate-producing bacteria was observed in IBD patients (87, 88). Evidence showed that *Faecalibacterium prausnitzii*, a major butyrate producer in the gut, may affect SCFAs production (87). The fecal level of SCFAs including butyrate and proprionate were lower in AS patients (89). Reduction in fecal medium chain fatty acids was also observed in patients with PsA compared to healthy controls (57).

Animal models showed that modulation of SCFAs could suppress systemic inflammation. Administration of SCFAs and fiber-rich diets could suppress lymphocyte driven systemic inflammation and ameliorate inflammatory arthritis (90, 91). Administration of SCFA propionate to HLA-B27/β_2_ microglobulin - transgenic rats also significantly reduced colonic inflammation and expression of inflammatory cytokines (92). The use of indole-3-acetic acid (microbial tryptophan metabolite) in proteoglycan-induced AS mice also ameliorated disease severity, dampened inflammatory response and restored the balance of gut microbiome composition (93). However, translational research on gut metabolites modulation in human is still lacking.

There are some challenges in gut metabolites research. Similar to studies on gut microbiome, gut metabolites are heavily influenced by genetic and environmental factors. Dietary intake of carbon and fibers have a strong deterministic effect on gut metabolites. Also, there is an interplay between gut microbiome and gut metabolites. Fluctuation in disease activity may result in dynamic variation in gut microbiome and metabolites. Standardization of sample collection and preparation methods is another key obstacle which may hinder the research development in gut metabolites. Future studies are required to broaden our understanding of microbial community and microbial metabolic pathways in SpA.

## Conclusion

SpA is a heterogenous group of diseases of overlapping phenotypes which involves the complex interplay between genetic and environmental factors. Genomic studies have increased our understanding on genetic predisposition in SpA. However, further research is required to translate these genomic findings into clinical practices for disease phenotyping and drug response prediction. Emerging evidence on gut-joint axis in SpA is promising and has shed new insights into the pathogenesis of SpA. Currently there is a huge knowledge gap regarding gut microbiomics and metabolomics in SpA. The development in this field is nascent but has been expanding rapidly. Future multi-omic studies may improve our understanding toward SpA and walk toward precision medicine by identifying new biomarkers and treatments.

## Author contributions

JS: manuscript preparation. A-SD, DE, and L-ST: critical comments and manuscript revision. All authors contributed to this article and approved the submitted version.

## Conflict of interest

The authors declare that the research was conducted in the absence of any commercial or financial relationships that could be construed as a potential conflict of interest.

## Publisher's note

All claims expressed in this article are solely those of the authors and do not necessarily represent those of their affiliated organizations, or those of the publisher, the editors and the reviewers. Any product that may be evaluated in this article, or claim that may be made by its manufacturer, is not guaranteed or endorsed by the publisher.
